# Advances in Reference Membranes for Potentiometric Sensing Applications

**DOI:** 10.3390/membranes15120376

**Published:** 2025-12-06

**Authors:** Martyna Drużyńska, Nikola Lenar, Beata Paczosa-Bator

**Affiliations:** Faculty of Materials Science and Ceramics, AGH University of Krakow, Mickiewicza 30, PL-30059 Krakow, Poland; druzynska@agh.edu.pl

**Keywords:** reference membranes, solid-state reference electrodes, potentiometric sensors, ionic liquids, miniaturized sensing platforms

## Abstract

Accurate potentiometric sensing critically depends on the stability and reproducibility of the reference electrode potential. Conventional liquid-filled Ag/AgCl or calomel electrodes, though well-established, are poorly compatible with miniaturized, portable, or long-term in situ sensing devices due to electrolyte leakage, junction potential instability, and maintenance requirements. Recent advances in solid-state and membrane-based reference electrodes offer a promising alternative by eliminating the liquid junction while maintaining stable and well-defined potential. This review summarizes the advancements in polymer-based and composite reference membranes, focusing on material strategies, stabilization mechanisms, and integration approaches. Emphasis is placed on ionic-liquid-doped membranes, conducting polymers, lipophilic salts, and carbon nanomaterials as functional components enhancing interfacial stability and charge transfer. The performances of various architectures, solid-contact, liquid-junction-free, and quasi-reference systems, are compared in terms of potential drift, matrix resistance, biocompatibility, and manufacturability. Furthermore, recent developments in printed, microfluidic, and wearable potentiometric platforms demonstrate how reference membrane innovations enable reliable operation in compact, low-cost, and flexible analytical systems. The review outlines current trends, challenges, and future directions toward universal, miniaturized, and leak-free reference electrodes suitable for innovative sensing technologies.

## 1. Introduction

Accurate potentiometric measurements rely fundamentally on the existence of a stable and well-defined reference potential against which the indicator electrode is measured. The reference electrode therefore influences the baseline and long-term reproducibility of potentiometric sensors: any fluctuation, drift or ill-defined potential of the reference directly translates into systematic error and degraded analytical performance. In conventional laboratory equipment, this function is commonly fulfilled by liquid-filled reference electrodes such as Ag/AgCl or saturated calomel, whose thermodynamically defined redox equilibria and internal electrolyte reservoirs provide a reproducible potential under standard conditions [1,2].

However, classical reference electrode designs exhibit significant limitations when adapted to miniaturized, portable or long-term in situ sensing environments. These electrodes need an internal electrolyte reservoir and a liquid junction to connect with the sample. This setup can cause unstable junction potentials, require refilling of the electrolyte, and make the internal electrolyte prone to dilution or drying out in small devices. Maintaining constant chloride activity (in Ag/AgCl systems), preventing salt leaching, and protecting against biofouling or contamination become challenging in small devices or complex matrices. These issues often cause greater signal drift, shorter lifetime, and more maintenance than traditional laboratory reference electrodes [3,4]. These challenges motivate the development of membrane-based, solid-contact, or quasi-reference electrode strategies that reduce or eliminate the need for a liquid internal electrolyte while retaining stable potential behavior. Membranes or polymeric matrices can perform multiple roles, serving as ion reservoirs that buffer local composition, acting as diffusion barriers limiting exchange with the external sample, and immobilizing salts or redox couples that define the reference potential. By utilizing polymer chemistry, ionic liquids, gel electrolytes, and conductive interlayers (such as carbon materials or conducting polymers), membrane-based approaches aspire to deliver compact, robust reference systems compatible with microfabrication, screen printing, and lab-on-chip integration. Recent studies report heterogeneous polymer membranes incorporating fixed salts, ionic-liquid-doped matrices, and leakless or bipolar reference configurations, each aiming to optimize trade-offs in short-term stability, long-term drift, matrix interference resistance, and manufacturability [3,5,6,7].

In this review, we present a comprehensive comparison of membrane-based reference electrode technologies, focusing on material strategies, architectural design, performance metrics, and integration paths relevant to miniaturized potentiometric platforms. We compare the leading design approaches, survey characterization methods for assessing stability and drift, and highlight examples where membrane-based references have enabled compact, field-ready potentiometric sensing.

## 2. Principles of Reference Membranes

Traditional reference electrodes such as Ag/AgCl and the saturated calomel electrode (SCE) remain the foundation for modern electrochemical measurements. Both systems rely on a well-defined redox couple (Ag/AgCl(s)/Cl^−^(aq) or Hg/Hg_2_Cl_2_(s)/Cl^−^(aq)) maintained in equilibrium with a stable internal electrolyte. Their potential is governed by the Nernst equation but remains effectively constant due to the fixed activity of chloride ions in the saturated electrolyte solution. These classical electrodes also incorporate a liquid junction containing a porous frit, which establishes ionic contact with the sample while minimizing junction potentials [8,9].

A related concept is the glass membrane electrode, where a hydrated glass layer forms a chemically selective phase boundary enabling stable potential generation. Although widely used for pH measurement rather than as reference electrodes, glass membranes provide a conceptual link to polymeric reference membranes, as both rely on controlled ion exchange and stable phase-boundary equilibria [10].

Polymeric and solid-state reference membranes replicate these principles, including fixed activity of internal ions, stable redox or ion-exchange equilibria, and predictable phase-boundary potentials, while eliminating the limitations of liquid-filled reservoirs and fragile frit junctions.

Reference membranes are engineered to provide a stable, quasi-invariant potential by maintaining a controlled ionic environment at the interface with the sample. The aim of such systems is to suppress undesired ion fluxes and fluctuations while ensuring that the reference potential remains insensitive to variations in bulk composition, pH, redox conditions or ionic strength. Understanding membrane-based reference electrodes therefore requires examination of two interrelated aspects: the mechanisms that establish and preserve stable potential, and the typical architectures that realize these mechanisms in miniaturized, solid-state formats.

### 2.1. Mechanism of Action

The mechanism by which a reference membrane maintains a stable potential is fundamentally governed by phase-boundary phenomena. In membrane-based reference systems, a reproducible potential arises from the controlled partitioning of ionic species between the membrane phase and the surrounding aqueous solution. When a polymeric membrane contains fixed ionic species—such as lipophilic or organic electrolytes, ionic liquids, or embedded salts—the equilibrium distribution of these ions across the interface determines the interfacial potential. The membrane composition, including the polymer type, nature and concentration of ionic additives, and the balance between hydrophobic and hydrophilic domains, directly influences the partition coefficients and thus the phase-boundary potential.

Recent theoretical and experimental studies have demonstrated that organic electrolyte additives and ionic liquids can stabilize this interface by internally compensating ionic activity, effectively creating a self-buffered phase boundary that is less sensitive to external ion concentration changes [11]. For instance, Kalinichev et al. [11] showed that polymer films doped with lipophilic electrolytes or ionic liquids exhibit enhanced stability because the partition equilibrium of these ions regulates the interfacial potential. Their work confirmed that such modifications reduce potential fluctuations even when the bulk electrolyte concentration varies.

Incorporating small amounts of a hydrophilic electrolyte (e.g., KCl) can further improve potential stability. These additives act as ion exchangers that moderate co-extraction between the membrane and the solution, thereby broadening the concentration range over which the reference potential remains constant [11]. Another effective stabilization strategy involves embedding a localized ionic reservoir within the membrane. The inclusion of sparingly soluble salts (e.g., KCl or other halides) or ionogel/gel-electrolyte phases provides a persistent source of ionic species near the electrode surface. This internal reservoir mitigates leaching or dilution effects that typically compromise the performance of miniaturized, liquid-filled reference systems. Numerous studies confirm that membranes containing solid salt inclusions or ionic liquid/plasticizer blends exhibit significantly lower potential drift compared to unbuffered solid contacts under varying sample conditions [3,12,13,14,15].

Among these strategies, the use of ionic liquids (ILs) has proven particularly effective. Zhang et al. [12] demonstrated that doping PVC membranes with the ionic liquid 1-methyl-3-octylimidazolium bis(trifluoromethylsulfonyl)imide and integrating a three-dimensionally ordered macroporous carbon solid contact produced liquid-junction-free reference electrodes with exceptional stability (drift ≈ 42 µV·h^−1^ over 26 days). This result validated the concept that a membrane with internally balanced ion partitioning can provide a stable reference potential without relying on a liquid junction.

Overall, partitioning-based stabilization depends on maintaining a quasi-closed system in which ion exchange with the environment is minimal. A membrane that is sufficiently hydrophobic and ion-impermeable, yet capable of supporting internal ionic buffering, resists external disturbances such as pH shifts, changes in ionic strength, or interfering ions. The combination of limited ion flux and robust internal buffering thus defines the long-term stability of modern reference membranes.

### 2.2. Typical Architectures

Membrane-based reference electrodes can be realized in several established configurations, each designed to balance compactness, stability, and ease of fabrication. The three most common types are solid-contact reference electrodes (SCREs), liquid-junction-free systems, and quasi-reference electrodes (QREs).

Solid-contact reference electrodes (SCREs) couple a conductive substrate (such as Ag/AgCl, noble metals, or carbon) with a membrane containing ionic additives or embedded salts, often through a conductive interlayer. This structure forms a compact, reservoir-free reference that is particularly suitable for planar or printed sensor platforms [1,14]. A representative example is the flexible sensor developed by Dam et al., where a PVC–KCl membrane was cast onto a screen-printed Ag/AgCl base, resulting in stable potential behavior without a separate liquid junction [16]. The elimination of internal electrolyte reservoirs not only simplifies miniaturization but also improves mechanical robustness and long-term sealing.

Liquid-junction-free reference electrodes go one step further by completely removing the classical salt bridge or porous frit. In these designs, the reference potential forms directly at the membrane/solution interface, which is defined by the ion partitioning equilibrium of the membrane components. Typically, polymeric membranes doped with organic electrolytes or ionic liquids act as self-contained junctions that maintain stable potential under buffered conditions. A paradigmatic study by Zhang et al. demonstrated that a PVC membrane doped with an ionic liquid (1-methyl-3-octylimidazolium bis(trifluoromethylsulfonyl)imide) and supported by a three-dimensionally ordered macroporous carbon (3DOM) contact exhibited remarkably low drift (~42 µV h^−1^ over 26 days) [12]. This design validated the possibility of achieving high stability without any liquid junction. Another liquid-junction-free electrode architecture was presented by Kalinichev et al. In this study, the PVC-based membrane was doped with organic solutions. The constructed reference electrode can be characterized by low potential drift (1 mV over 24 h) [11].

Solid-contact reference electrodes and liquid-junction-free reference electrodes are not equivalent, although both eliminate the classical porous liquid junction. Solid-contact reference electrodes are fully solid-state devices in which a membrane is deposited directly onto a conductive substrate. Liquid-junction-free reference electrodes simply remove the traditional liquid junction but may still incorporate an internal electrolyte phase, e.g., liquid, gel, ionic liquid, or polymer electrolyte. These electrodes do not always rely on a membrane adsorbed onto a conductive material.

Quasi-reference electrodes (QREs) represent a simplified, pragmatic approach for short-term or disposable applications. In such systems, absolute potential stability is not strictly maintained, but moderate drift is acceptable within the measurement timeframe. Carbon-based materials (graphite, carbon paste) or conducting polymers are often employed as QREs, typically protected with a thin polymer or ionogel layer. These electrodes are advantageous due to their low cost and simple fabrication, though they exhibit higher drift and lower reproducibility compared to buffered or junctionless membrane systems [3,17].

More advanced designs employ multilayer architectures, in which a base metal (e.g., Ag or Ag/AgCl) is successively overcoated with functional layers such as conducting polymer interlayers (e.g., PEDOT:PSS), ionogel or ionic-liquid layers, and outer diffusion-control membranes. These layered configurations enhance mechanical durability, reduce leaching of internal ions, and provide an additional barrier against external chemical interference [1].

Beyond chemical design, electrical and mechanical optimization is equally critical. The integration of high-capacitance solid contacts—such as conducting polymers, three-dimensionally ordered carbon, or carbon nanotube networks—enhances charge storage at the electrode/membrane interface, reducing potential fluctuations from transient currents or environmental perturbations. Such interlayers provide a stable, electronically conductive link between the sensing membrane and the measurement circuit, enabling robust signal readout in fully solid-state potentiometric systems [3,13].

To illustrate the diversity of current reference membrane architectures, Table 1 summarizes representative examples reported in the recent literature. The table compares structural types, membrane compositions, stabilization mechanisms, potential stability metrics, and typical applications. These examples highlight how chemical design (e.g., ionic liquids, lipophilic electrolytes, ionogels) and physical architecture (solid contact type, layered structure) together determine the performance and applicability of reference membranes in potentiometric sensors.

Each design has its pros and cons—it balances short-term stability, long-term changes over time, sensitivity to environmental factors (like pH, salt levels, or other interfering substances), as well as how easy it is to make and how safe it is for biological use. For example, ionic-liquid-doped membranes can provide excellent leak-free behavior and low drift, but the choice of plasticizers and ILs must account for potential leaching and biocompatibility constraints in wearable or implantable applications [15,17]. Likewise, embedding solid salts improves buffering but requires careful control to avoid phase separation, recrystallization or mechanical failure during device fabrication and use [1].

## 3. Materials and Design Approaches

Polymer-based membranes are typical design solutions for all-solid-state reference electrodes. The composition of such a membrane contains polymers (e.g., PVC, PU) and plasticizers (DOS, o-NPOE) [1,18,19]. Such membranes are a promising base for modifications using advanced materials. The main components used for designing polymer-based membranes and their function are presented in Table 2.

In this section, typical design approaches and commonly used modifiers for polymeric membranes are described. The influence of conducting polymers, carbon nanomaterials, ionic liquids, and lipophilic salts used as membrane additives are investigated in Section 3.1, Section 3.2, Section 3.3 and Section 3.4. Section 3.5. is dedicated to composite membranes.

### 3.1. Conducting Polymers

Materials with ion-exchanging and redox properties can be successfully used as solid-contact layers for all-solid-state electrodes. It is proven that using conducting materials as an intermediate layer enables obtaining a stable electrode potential [23].

**Figure 1:**
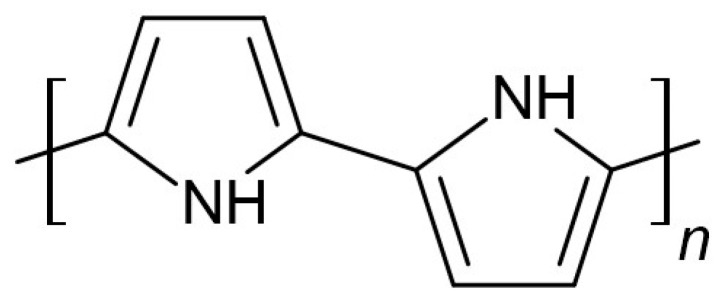
Polypyrrole structure.

A. Kisiel et al. [24] presented polypyrrole (Py) (Figure 1) as an ion-to-electron transducer for solid-contact reference electrodes. The proposed cell contains reference and indicator electrodes with poly(n-butyl acrylate)-based membranes covering a conducting polymer layer. The polypyrrole layer was doped with chloride ions, then electrodeposited on glassy carbon (GC) electrodes. A membrane cocktail for the reference electrode was prepared by mixing tetradodecylammonium tetrakis(4-chlorophenyl) borate (ETH 500), AgCl (with metallic Ag), KCl, n-butyl acrylate, and a crosslinking mixture containing 1,6-Hexanediol diacrylate (HDDA) and 2,2-dimethoxy-2-diphenylacetophenone (DMPP) then cast onto the GC electrode with the polypyrrole layer. After that, photopolymerization was carried out. Before measurements, the electrodes were conditioned overnight in 3 M KCl solution.

In order to examine signal stability and potential interferences, potentiometric measurements were carried out in 10^−5^ to 10^−1^ M solutions of KCl, NaCl, and NaNO_3_. The results have shown that the potential is practically independent of anion or cation kind and salt concentration; the absolute values of the slopes are lower than 1 mV per decade. To investigate potentiometric signal indication in different conditions simulating real-life samples, potential depended on changing pH and redox conditions as well as bubbling sample with oxygen and nitrogen and surfactant influence. The constructed reference electrodes are practically independent of kind and concentration of electrolyte solutions, and the influence of typical interferences such as oxygen, changing pH, redox couples’ presence, and surfactants is low. The presented electrodes are promising and robust tools for analysis.

**Figure 2:**
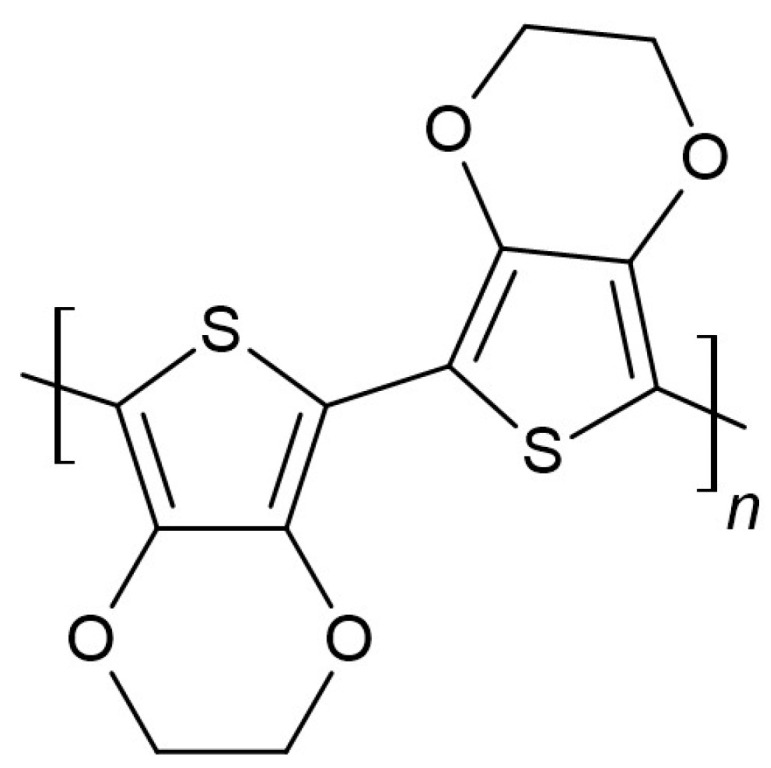
Poly(3,4-ethylenedioxythiophene) structure.

Poly(3,4-ethylenedioxythiophene) (PEDOT) (Figure 2) is another kind of conducting polymer commonly used as a solid-contact layer. The conducting polymers in solid-contact layers act as ion-to-electron transducers. This approach was presented by C. Zuliani et al. [13]. In order to prepare reference electrodes, conducting polymers poly(3-octylthiophene-2,5-diyl) (POT) and PEDOT were deposited onto carbon-printed electrodes to form the intermediate layer. Prepared electrodes with a solid-contact layer were rinsed with ethanol and water and dried by blowing nitrogen over them. The capping membranes consisted of the ionic liquid (e.g., [emim][FAP], [emim][NTf2]), the acrylate monomer, the crosslinker, linking acrylate monomers (e.g., 1,6-hexanediol diacrylate (HDDA)), and the photoinitiator, initiating polymerization (e.g., 2,2-dimethoxy-2-phenylacetophenone (DMPP)).

The experiments conducted have shown that changes in the potential of the electrode were found to be relatively small in varying concentrations of background ions (Na^+^ and K^+^). These results indicate that the designed reference electrode exhibits very minor potential variations with changing ionic strength, confirming that it behaves as a stable and reliable reference.

### 3.2. Ionic Liquids

Increasingly popular additives are ionic liquids (ILs). They can be defined as organic salts that are liquid below 100 °C. Because of their properties such as nonflammability and low volatility, they are the alternative for organic solvents and fit into the foundation of green chemistry [25,26]. When replacing a conventional reference electrode with an ionic-liquid-based reference electrode, researchers must consider some criteria such as the purity of the ionic liquid, implementation of the ionic liquid electrode into the membrane, composition of the membrane, and experimental conditions under which the electrode will be used [27].

**Figure 3:**
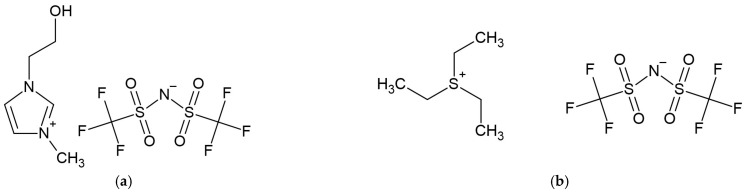
(**a**) 1-(2-hydroxyethyl)-3-imidazolium bis{(trifluoromethyl)sulfonyl}imide, (**b**) (triethylsulfoniumbis{(trifluoromethyl)sulfonyl}imide bis(trifluoromethylsulfonyl)imide) structures.

J. Kuczak et al. [5] investigated the influence of physicochemical properties of ionic liquids on the performance of polymer-based membrane reference electrodes. Membranes were prepared by dissolving different ionic liquids such as 1-(2-hydroxyethyl)-3-imidazolium bis{(trifluoromethyl)sulfonyl}imide (Figure 3), triethylsulfonium bis{(trifluoromethyl)sulfonyl}imide bis(trifluoromethylsulfonyl)imide in polyurethane, and 2-nitrophenyl octyl ether (o-NPOE) polymeric matrix. Membranes were cast onto the surface of silver and glassy carbon-disk electrodes with a PEDOT/PSS intermediate layer. Before measurements, reference electrodes were conditioned in 10^−2^ M KCl solution. In order to examine physicochemical properties of IL, hydrophobicity tests were conducted. Potential stability tests were performed in NaClO_4_ and TMACl aqueous solutions of changing concentrations. A long-term stability test was conducted in the solution of Na^+^, K^+^ and Cl^−^ imitating biomedical samples. The results have shown that 1-(2-hydroxyethyl)-3-imidazolium bis{(trifluoromethyl)sulfonyl}imide induced the best potential stability of reference electrodes. While examining different membrane compositions, it was stated that the amount of the ionic liquid in the membrane does not significantly influence analytical performance; it only depends on physiochemical properties of used IL.

**Figure 4:**
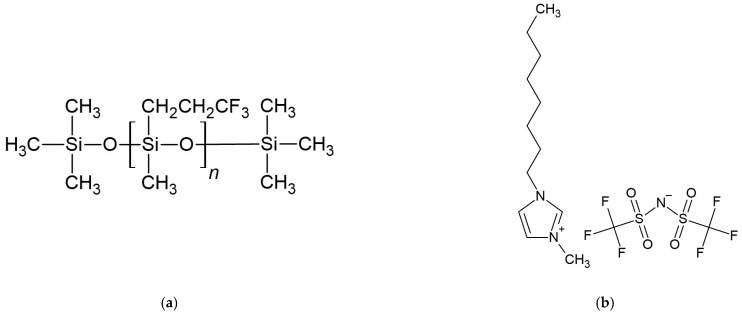
(**a**) poly(3,3,3-trifluoropropylmethylsiloxane), (**b**) 1-methyl-3-octylimidazolium bis(trifluoromethylsulfonyl)imide structures.

X. V. Chen et al. presented a slightly different approach [15]. Solid-contact reference electrodes were prepared using planar gold electrodes embedded in a polymer shaft. The solid-contact electrodes were prepared using CIM carbon as a transducer layer. A silicone membrane containing poly(3,3,3-trifluoropropylmethylsiloxane) (Figure 4a) doped with 1-methyl-3-octylimidazolium bis(trifluoromethylsulfonyl)imide ([C8mim+] [NTf2-]) (Figure 4b) was applied onto the gold electrode surface. The electrodes were conditioned in a 10^−3^ M KCl solution for two days before use. The presented reference electrode provided a stable potential registered in different concentrations of KCl. Over 8 days in artificial blood electrolyte solutions, the reference membranes exhibited a potential drift as low as 20 µV/h.

### 3.3. Carbon Nanomaterials

Carbon nanomaterials are widely used in the design of potentiometric sensors. They are characterized by chemical stability, high specific surface area, and good electrical conductivity, which makes them suitable for use as solid-contact layers [28]. Different kinds of carbon nanostructures could be used as intermediate layers, for example, graphene [29,30], fullerenes [31], or carbon nanotubes [32].

F. X. Rius-Ruiz et al. [3] introduced single-walled carbon nanotubes (SWCNTs) as a solid-contact layer for the reference electrode. Carboxylated SWCNTs were deposited onto the surface of the previously polished GC electrode. Then, the obtained intermediate layer was covered with the reference membranes containing photo-polymerized n-butyl acrylate polymer, crosslinking mixture, KCl, AgCl with traces of Ag, and ETH500. Reference electrodes were conditioned for 24 h on 3 M KCl solution. In order to examine potential stability, the potentiometric response in NaCl, KCl, and NaNO_3_ solutions was measured. The influence of changing pH conditions was also investigated. The electrodes exhibit sensitivity in the range of 0.5–1 mV/dec. There were no significant changes in potential from pH 3 to 10. The experiments have shown that designed reference electrodes are characterized by stable potential.

### 3.4. Lipophilic Salts

Lipophilic salts are a common additive to ion-selective membranes as well as to membranes used in reference electrodes. It is proved that presence of lipophilic salts in the membrane provide stable and reproducible electrode potentials [33].

**Figure 5:**
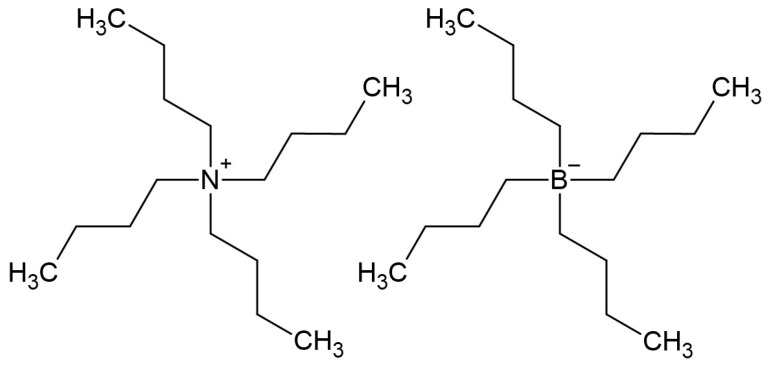
Tetrabutylammonium Tetrabutylborate structure.

U. Mattinen et al. [34] demonstrated the effect of adding salt to the membrane of a solid-contact reference electrode. Reference electrodes were prepared by drop casting membranes on two different substrates: glassy carbon (GC) disks covered with the conductive polymer doped with chloride (PEDOT-Cl) and silver disks coated with silver chloride. Lipophilic salts were introduced into the plasticized PVC membrane. Two approaches were demonstrated. First, the effect of single salt electrodes (TBA-TBB) (Figure 5) in the membrane was examined. The results have shown that single salt electrodes in the membrane resulted in good potential stability but slow electrode response. Then, the effect of double salt in the membrane was examined. This effect is based on a reaction between organic salts added into the membrane. Two salts (i.e., TBACl + KTPhB) reacted with each other to form organic salt and KCl. It was found that this approach demonstrated improved insensitivity to concentration changes when comparing the membranes containing corresponding single salt only.

Lipophilicity criteria are very important in terms of designing reference membranes. N. Tiuftiakov et al. investigated this phenomenon by developing a new type of RE based on membrane doped with lipophilic salts and ionophores. Usually, very lipophilic salts might cause unstable potentials due to ion-composition mismatch. Incorporating ionophore into a polymeric membrane creates a stable potential through combined ion complexation. The designed membrane contains poly(vinyl chloride), 2-nitrophenyl decyl ether (NDPE) as a plasticizer, valinomycin as a potassium selective-ionophore, and various lipophilic salts (e.g., KTpClPB). The authors verified the mechanism with theoretical simulations and by conducting potentiometric measurements. The obtained reference electrodes have shown stability in KCl solutions, with very small potential drift (~0.4 mV) [35].

### 3.5. Composite Membranes

The main objective of designing composite membranes is to construct sensing devices mechanically robust with developed analytical performance [36]. In terms of fabricating reference electrodes, linking polymer with inorganic salt (e.g., KCl) is quite common [37]. T. Guinovart et al. [38] presented a polymeric membrane reference electrode using a polyvinyl butyral (PVB) matrix, where Ag/AgCl and NaCl were developed for decentralized chemical measurements. To prepare an electrode, NaCl and AgNO_3_ are added to the stock solution and homogenized, forming a white AgCl colloid. The colloid is then briefly exposed to light to partially reduce AgCl to Ag. Afterward, the suspension is kept in the dark, drop cast onto a polished glassy carbon electrode, and dried. Before use, the dried electrode is conditioned in KCl to minimize sensitivity to changes in ion concentrations. The electrode presented by T. Guinovart et al. is characterized by a stable potential (less than 1 mV dec^−1^) over a wide range of concentrations for the several chemical species (such as LiCl, NH_4_Cl, MgCl_2_, Na_2_SO_4_, NaHCO_3_). Moreover, no significant influence to changes in redox potential, light, and pH are observed.

D. S. Macedo et al. [39] presented a solid-state reference electrode based on poly(vinyl acetate)/KCl composite. The electrode was prepared by mixing solid KCl with a liquid monomer containing EGDMA as a crosslinker to form a thick slurry. The obtained mixture was pipetted into small glass vials, and a silver wire coated with AgCl was immersed in the slurry. The next step was polymerization of the monomer. After that, the bottom of each vial was ground off to expose the solid composite material, which served as the contact surface of the electrode. Conducted laboratory tests have shown that incorporating a crosslinked polymer demonstrated a stable reference potential for 175 days in 1 M Na_2_SO_4_ solution. When used together with a commercial glass pH electrode, the solid-state reference electrode maintained a consistent potential across different pH buffer solutions, enabling accurate pH measurements in various media. Moreover, electrodes showed a reduced rate of KCl leakage, suggesting that crosslinking may extend the electrode’s operational lifetime.

A. Lewenstam et al. [40] designed a 3D-printed electrode with PVC/KCl composite acting as the electroactive element. Highly stabilized and plasticized PVC/KCl mixtures were prepared by combining PVC, Ca/Zn as a heat stabilizer, and diethylhexyl terephthalate (DETH) as a plasticizer with the dispersed KCl powder. The resulting materials had a defined hardness and were formed into cylindrical filaments by repeatedly applying controlled temperature and pressure. The complete reference electrode was fabricated by 3D printing an acrylonitrile butadiene styrene (ABS) base, depositing a silver/silver chloride layer, and then completing the system by 3D printing the previously prepared electroactive composite material on top. Before measurements, the electrode was conditioned for 1 h in 0.1 M KCl. The SCREs demonstrated stable potential across varying KCl concentrations. They maintained stable potential readings even after three months of immersion in 0.1 M KCl, indicating excellent long-term durability. The electrodes were also unaffected by changes in redox conditions. Moreover, during strong acid-based titrations, the SCREs also retained stable potential.

## 4. Performance and Characterization

To be a robust tool in electrochemical analysis, reference electrodes should meet specific requirements. Reference electrodes must exhibit a stable and well-defined potential that does not change significantly over time or with variations in the composition of the test solution. Moreover, when redox mediators are added to the membrane, reference membranes should provide a reversible redox couple with a fast electron transfer rate to ensure accurate measurements [41,42].

### 4.1. Potential Stability

Potentiometric response given by a reference electrode should be well-defined and stable regardless of changing conditions. Any registered instability directly affects results in chemical analyses and could lead to incorrect determination. In the process of time, some changes in potential values can be observed. Desirable potential drift should be as low as possible. Depending on application, the reference electrode can be successfully useful if the potential drift value is lower than 1 mV/day [43,44]. Increasing drift might be caused by changes in the activity of the ions in the electrolyte or electrode’s component degradation [45].

### 4.2. Chemical Robustness

Reference electrodes should exhibit steady potential and unchanged electrical properties while being influenced by chemical factors such as changing pH values or interferents presence [46]. For example, electrode structures that include silver wire can be prone to corrosion caused by exposure to sulfides. That is why it is crucial to identify potential interferents and adjust electrode construction while developing new architecture and functional materials [14,47]. Acceptance criteria for reference electrode usefulness are low potential drift after exposure to damaging factors and no significant loss of electrode material [46,48].

Apart from chloride-driven dissolution, many other chemical species can compromise the stability of Ag or Ag/AgCl layers used as internal conductors in reference electrode structures. Certain anions such as perchlorate (ClO_4_^−^) or thiocyanate (SCN^−^) can penetrate polymer layers and disrupt the Ag/AgCl equilibrium by forming alternative surface complexes. Redox-active molecules (such as ascorbate, uric acid, Fe^2+^/Fe^3+^) may reduce or oxidize surface silver species, leading to undesired shifts in potential. Strong ligands and complexing agents, including cyanide, ammonia, or thiosulfate, can dissolve silver by forming highly stable soluble complexes.

### 4.3. Techniques for Evaluation

There are several methods, adapted for electrode testing, which allow for efficiency and performance evaluation. Those techniques enable us to predict electrode performance with laboratories and real-life samples. In addition to methods that allow for the analysis of electrode surfaces after conducted tests (such as SEM, EDS), there are methods that provide direct information on changes in potentiometric response.

The Open Circuit Potential technique is based on potential measurements in standard solution with no current flow. Changes in potential indications can be registered under different conditions (i.e., changing temperature, pH, ion concentration) and inform about potential drift [4,44].

Changes in potential can be registered under conditions with forced current flow; here, chronopotentiometry finds its application. This method provides information on the resistance of reference electrodes to polarization and on the quality of the contact between the solid-contact layer and the solution [4,49].

Impedance measurements over a wide frequency range are possible using Electrochemical Impedance Spectroscopy. The most important information provided by this method is the evaluation of the resistance of the solution, the contact layer, and the stability of the electrode structure. Comparison of EIS results before and after prolonged exposure to the test environment (for example, exposure to corrosion factors) allow for the assessment of the direct impact of factors on the performance of the reference electrode [4,50].

In addition to potentiometric drift measurements, cyclic voltammetry (CV) may also be employed to assess reference electrode stability. By recording CVs of a well-defined reversible redox couple, such as [Fe(CN)_6_]^3−^/[Fe(CN)_6_]^4−^, the half-wave potential (E_1_/_2_) can be monitored as an indirect indicator of reference electrode potential. A stable reference electrode produces reproducible E_1_/_2_ values over repeated scans or over time, while shifts in E_1_/_2_ reflect changes in the reference electrode potential. Although CV does not replace long-term OCP drift tests, it serves as a useful complementary method for rapid screening of membrane formulations or detecting short-term instability [51,52].

A summary of the functionalities of the methods presented is shown in Table 3.

Because of the search for new materials and various membrane formulations, there is no specific protocol for reference electrode evaluation. However, there are some criteria that should be met to consider the newly designed reference electrode useful. Potentiometric measurements should include calibrations—to check the slope of the response is Nernstian. Another criterion is potential stability. This property should be evaluated over both short and long time scales. The reproducibility should be tested; it would provide information about any differences in signal indications between different items prepared using the same membrane formulation. Another operational parameter should include working pH range, temperature range, minimum and maximum immersion times, and compatibility with different external electrolytes.

## 5. Application: Integration into Sensing Platforms

Integration of membrane-based reference electrodes into diverse sensing architectures has become essential for modern potentiometric systems. Compact, leak-free, and stable references enable reliable operation across printed, microfluidic, multi-sensor, and wearable devices. The following subsections summarize representative examples and illustrate the practical use of these technologies.

### 5.1. Miniaturized and Printed Electrodes

Printed technologies, including screen printing and inkjet printing, enable low-cost fabrication of miniaturized reference electrodes on flexible or disposable substrates. Screen-printed electrodes (SPEs) are especially attractive due to their compatibility with mass production and their robust analytical performance [53]. A landmark demonstration was reported by Tymecki et al., who produced fully screen-printed Ag/AgCl/KCl reference electrodes on polyester substrates. Their multilayer structure, consisting of an Ag/AgCl conductor, a KCl-doped polymer electrolyte, and a UV-cured protective coating, was fabricated with no manual post-treatment and exhibited excellent long-term stability (drift < 0.2 mV h^−1^) over 10 days of continuous immersion [54]. This work established a scalable route for producing reliable reference elements within fully printed potentiometric systems.

Further developments expanded this concept to various substrate materials and membrane chemistries. Hayat et al. demonstrated that disposable printed Ag/AgCl reference layers protected with polymeric coatings can deliver stable performance in environmental sensors, especially when combined with screen-printed indicator electrodes [53]. In a three-electrode potentiometric setup, the indicator electrode develops the analyte-dependent potential, the reference electrode provides a stable and constant comparison potential, and the auxiliary electrode simply completes the circuit but remains inactive because no current flows during potentiometric measurements. An example application of the miniaturized platform was presented by Ruecha et al. The researchers reported a fully inkjet-printed paper-based device integrating both ion-selective and reference elements for Na^+^ and K^+^ detection; this work proved that low-cost, solvent-free printing can produce functional potentiometric systems with stable potential over several hours [55]. In addition, Dawkins et al. evaluated the long-term stability of screen-printed Ag/AgCl references, showing that proper ink formulation and surface conditioning significantly reduced drift in continuous immersion tests [56].

**Figure 6:**
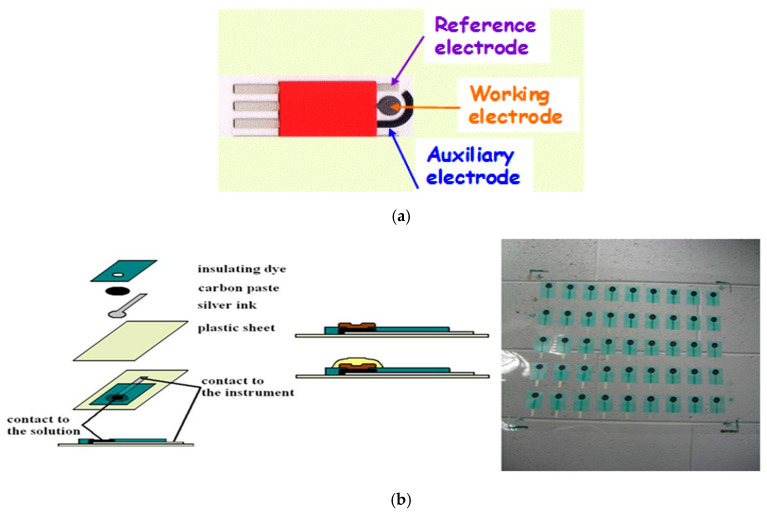
Miniaturized and printed electrodes: (**a**) disposable screen-printed electrochemical sensors for environmental monitoring [53]; (**b**) screen-printed platforms for indicator and reference electrodes—reprinted with the permission from John Wiley and Sons reference [57].

Several groups also explored polymer-based or ionic-liquid-modified membranes that function as liquid-junction-free printed reference electrodes. Cicmil et al. introduced ionic-liquid-doped PVC membranes compatible with screen printing, which showed excellent potential stability during potentiometric titrations and were resistant to changes in sample composition [57]. Rius-Ruiz et al. developed a planar reference electrode comprising a polyacrylate membrane with carbon nanotubes, demonstrating low-cost fabrication and stable potentials suitable for on-site use [58]. Kisiel and colleagues expanded the family of printable reference membranes by introducing poly(n-butyl acrylate) compositions that improved membrane flexibility and adhesion to printed platforms [20]. Together, these studies illustrate progression towards fully integrated, miniaturized, and mechanically robust reference electrodes suitable for disposable or flexible potentiometric devices. The construction solutions of the electrodes related to miniaturization and printing are shown in Figure 6.

### 5.2. Microfluidic and Lab-on-Chip Systems

Microfluidic devices operate with small sample volumes and restricted ion reservoirs, making reference electrode stability particularly challenging. Safari-Mohsenabad et al. developed microfluidic reference electrodes with free-diffusion liquid junctions that minimized potential fluctuations while providing rapid response in flowing streams [59]. Their architecture demonstrated that controlled diffusion interfaces can support stable on-chip potentiometry.

Alternative strategies rely on solid-contact mechanisms that eliminate the need for liquid junctions altogether. Zuliani et al. implemented a PEDOT:PSS solid contact capped with an ionogel layer, creating a compact, liquid-junction-free microfluidic reference electrode with drift below 0.5 mV day^−1^ [13]. Building on this idea, Guagneli et al. integrated a solid-contact reference into a planar flow-through potentiometric sensor, demonstrating accurate real-time ion analysis in environmental and process monitoring applications [60]. Hu et al. expanded solid-contact approaches to disposable paper-based devices, using colloid-imprinted mesoporous carbon and a PVC membrane to achieve low drift and reproducible potentials suitable for low-cost microfluidic platforms [61]. In addition, Mattinen et al. showed that lipophilic salt-based polymeric membranes can sustain long-term stability with minimal ion leakage, making them particularly well-suited for confined microfluidic environments with limited electrolyte reservoirs [34]. Overall, these studies demonstrate how polymeric and solid-contact reference materials enable the reliable operation of integrated microfluidic and lab-on-chip systems.

**Figure 7:**
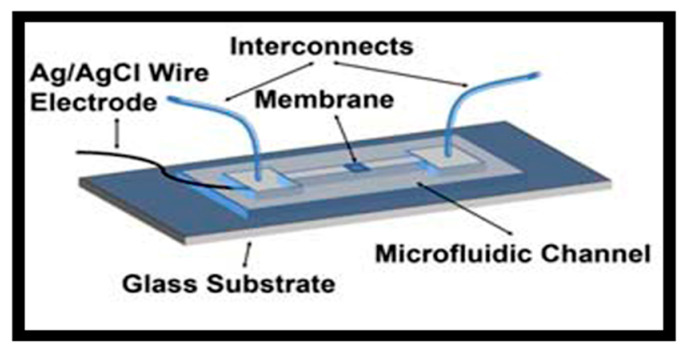
The solution illustrates microfluidic and lab-on-chip systems: microfluidic reference electrodes with free-diffusion liquid junctions [59].

The solutions illustrating microfluidic and lab-on-chip systems are shown in Figure 7.

### 5.3. Environmental Analysis

Environmental monitoring requires reference membranes that withstand variable ionic strength, fluctuating temperatures, and potential biofouling while maintaining stable potentials. Many printed reference electrodes have demonstrated suitability for field use. Hayat and Marty highlighted their durability in water quality sensing, particularly when protected with polymer coatings that limit interference from natural matrices [53]. Baumbauer et al. further demonstrated that screen-printed nitrate sensors incorporating planar solid-state reference electrodes can be deployed directly in soil and water samples, enabling distributed, real-time ion analysis under environmental conditions [62]. These developments show that printed and solid-state reference membranes can support continuous or in-field measurements across diverse environmental matrices.

**Figure 8:**
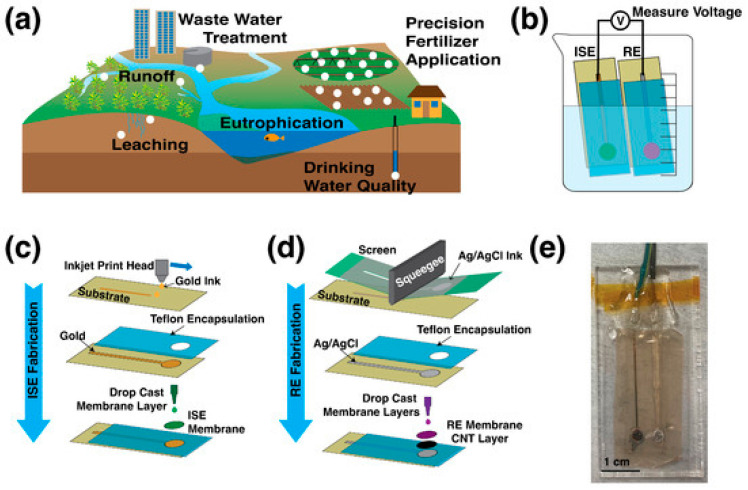
Printed nitrate sensors for soil and water applications, integrating a planar solid-state reference electrode: (**a**) illustration of nitrate contamination pathways in soil and aquatic environments, including runoff, leaching, and eutrophication; (**b**) schematic measurement setup; (**c**) fabrication process of the printed nitrate ISE; (**d**) fabrication process of the solid-state reference electrode; (**e**) photograph of the assembled printed nitrate sensor [62].

The scheme of constructed sensors is presented in Figure 8.

### 5.4. Biological and Medical Analysis

In biological and medical contexts, reference membranes must be biocompatible, mechanically flexible, and resistant to biofouling or electrolyte loss, as these conditions often involve low ionic strength and complex physiological matrices. Cuartero and Crespo highlighted that solid-contact and ionic-liquid-based reference membranes offer an optimal balance of flexibility, mechanical integrity, and potential stability for wearable potentiometric sensors designed to monitor ions in sweat or saliva [63,64]. Extending these concepts toward implantable applications, Chen et al. developed silicone-based reference membranes doped with ionic liquids that demonstrated stable operation in physiological media without electrolyte leakage or cytotoxic effects. Their system achieved exceptionally low drift (~20 µV h^−1^ over 8 days at 37 °C in artificial blood electrolyte), confirming its suitability for long-term monitoring in biomedical environments [15].

A further significant contribution was presented by Gan et al., who introduced a solid-contact reference electrode (SC-RE) based on the silver/silver tetraphenylborate (Ag/AgTPB) system, specifically engineered for flexible and wearable potentiometric platforms [14]. Their design employs a silver substrate coated with a thin AgTPB layer, followed by a PVC membrane plasticized with NPOE and containing the hydrophobic electrolyte tetrabutylammonium tetraphenylborate (TBATPB). By replacing the traditional hydrophilic salt bridge with a hydrophobic, fully solid configuration, the electrode eliminates electrolyte leakage and improves mechanical robustness. The Ag/AgTPB/PVC–TBATPB system provides a stable and reproducible potential across ionic strengths ranging from 10^−5^ to 10^−1^ M NaCl, while remaining insensitive to redox-active species, light, or dissolved gases. Importantly, the device maintained its functionality under repeated bending up to 90°, owing to the flexible PET substrate and PDMS insulation layer. When integrated with a chloride-selective electrode, the SC-RE enabled reliable sweat–chloride measurements during human exercise, demonstrating that the hydrophobic Ag/AgTPB interface combined with a polymeric reference membrane forms a robust and durable reference suitable for wearable and long-term potentiometric sensing [14].

To illustrate the diversity of sensing contexts in which membrane-based and solid-contact reference electrodes are employed, Table 4 summarizes representative analytes detected using potentiometric platforms discussed throughout this section. These examples highlight the broad applicability of such reference systems across environmental, biomedical, wearable, and printed sensor technologies and demonstrate how stable reference membranes enable reliable measurements across a wide range of ionic and chemically challenging sample matrices.

## 6. Challenges and Future Perspectives

Despite significant advances in membrane-based reference electrodes, several challenges remain before they can fully replace conventional references in demanding applications. In this section, we discuss three major issues, long-term stability, standardization, and recalibration, and emerging material strategies including hybrids, MOFs, and structural ions.

### 6.1. Long-Term Stability

Long-term stability is arguably the most critical hurdle for reference membranes. Drift over weeks to months, due to slow leaching of ions, membrane degradation, hydration/dehydration cycles, or chemical contamination, undermines the reliability of potentiometric measurements in real-world deployment. In a notable study, Blidi et al. evaluated solid-state composite (SSC) reference electrodes over 12 to 25 weeks of continuous use. They found that, after appropriate conditioning, their SSC electrodes maintained reproducibility within about ±1 mV and acceptable performance in calibration protocols across multi-solution tests [4]. Such results are among the best reported to date, but they still underscore that even advanced membrane systems require careful conditioning and may drift under challenging conditions.

Another challenge is poisoning or interference from species in complex matrices—for example, sulfide ions can degrade Ag/AgCl reference systems. A recent study demonstrated a sulfide-resistant solid-state reference electrode, using sacrificial AgCl in a crosslinked polymer matrix, and showed stable potential for ~120 days even in 1 g·L^−1^ Na_2_S solution, significantly extending the useful lifetime in harsh environments [47]. This illustrates how clever design (sacrificial buffering, protective matrices) can extend lifetime, but such strategies must be tested broadly across relevant media.

Temperature fluctuations, mechanical stress, and exposure to biofouling or chemical contaminants also accelerate degradation. Thin-film or ultrathin reference electrodes often suffer more from environmental perturbations and mechanical delamination [65]. Ensuring stable interfacial adhesion, minimizing water ingress, and selecting chemically robust polymer matrices remain key to mitigating long-term drift.

Looking ahead, long-term stability may benefit from self-rejuvenating or regenerative membranes (e.g., embedding reservoirs of buffer salts, reversible ionic reservoirs) or dynamic control of membrane composition. Coupled with real-time drift correction techniques (e.g., periodic internal recalibration), these strategies could push membrane references toward lifetimes rivaling classical liquid junction electrodes.

### 6.2. Standardization and Recalibration

A major obstacle to wider adoption of membrane-based reference electrodes is the lack of well-established standards for performance evaluation, calibration protocols, and drift compensation. In contrast, classical reference electrodes have decades of conventions for measuring junction potentials, salt bridge design, and calibration in known ionic media.

Membrane references require new standardization: for example, defining a set of test solutions (varied ionic strength, pH, interfering ions) and protocols (multi-solution cycling, long-term drift tracking, temperature cycles) to compare across laboratories. The SSC electrode study by Blidi et al. employed a ”multi-solution protocol” (MSP) to systematically assess drift and robustness across ionic strength, pH, and different ion species [4]. Wider adoption of such standardized protocols would enhance reproducibility and comparison.

Recalibration is another challenge: membrane systems may require periodic recalibration to correct for residual drift. Embedding internal calibration standards (e.g., small compartments with fixed salt solutions or microfluidic calibration channels) or designing dual-reference schemes (one stable, one sacrificial) may help. Another possibility is periodic “self-calibration” via exposure to reference solutions (e.g., KCl) or internal redox buffers.

Developing a consensus on recalibration intervals, acceptable drift thresholds, and methods to monitor drift in situ (e.g., monitoring potential shifts relative to an internal stable marker) will be critical for integrating membrane REs into field-deployable systems.

### 6.3. New Materials

To overcome limitations of existing polymer/ionic liquid membranes, emerging material strategies hold exciting promise. Hybrid materials—combining polymers, inorganic fillers, conductive nanomaterials (graphene, carbon nanotubes, metal oxides) and ionic liquids—can synergistically balance ion blocking, mechanical robustness, ionic buffering, and conductivity.

Metal–organic frameworks (MOFs) are especially intriguing: their high porosity, tunable pore environments, and chemical modifiability allow controlled ion transport and selective filtering. Although MOFs are more often studied in energy storage or catalysis, recent reviews describe progress in MOF composites for solid electrodes and ionic conduction applications [66]. Embedding MOFs within polymer matrices for reference membranes could allow precise control over ionic pathways, limiting leakage and improving stability.

Immobilized ionic groups, that is, ions covalently incorporated into the polymer backbone or crosslinkers rather than mobile dopants, represent another promising design direction. Polymers containing such fixed anionic or cationic sites, or coordination motifs that bind ions within the matrix, can minimize leaching and help maintain internal ionic balance. Combining these immobilized charges with dynamic ionic reservoirs may lead to membranes with intrinsically stable potentials [67].

Another promising avenue is stimuli-responsive membranes: materials whose ionic conductivity or permeability shifts with temperature, pH, or applied potential, enabling adaptive control of the reference environment. For example, embedding thermoresponsive gels or pH-responsive ionic polymers may help buffer potential under changing conditions [68].

Ultimately, the development of next-generation reference membranes will likely rely on multimodal materials: hybrids combining ionic liquids, MOFs, structural ions, highly conductive interlayers, and self-healing polymer networks. To support translation, these new materials need rigorous testing—drift over months, cycling, bio/chemical compatibility, and manufacturability—and must integrate with calibration and monitoring strategies.

## 7. Conclusions

Membrane-based reference electrodes are transforming how potentiometric sensors are designed and used. By replacing the traditional liquid-filled reference with solid, polymer-based, or hybrid membranes, researchers have achieved electrodes that are smaller, more stable, and easier to integrate into modern devices. Materials such as ionic liquids, conducting polymers, and carbon nanomaterials play a key role in improving signal stability and long-term performance.

Among the different designs, solid-state and liquid-junction-free systems stand out for their ability to deliver reproducible potential without maintenance or electrolyte leakage. These advances make reference electrodes compatible with flexible, wearable, and disposable sensor platforms—opening the door to reliable, real-time chemical and biological monitoring outside the laboratory.

Despite the progress, a few challenges remain. Reference membranes still need better resistance to long-term drift and environmental changes, as well as improved biocompatibility for medical applications. Future efforts will likely focus on developing self-buffered, composite membranes with built-in ionic reservoirs and on adapting scalable fabrication methods such as screen or inkjet printing.

In summary, research in this area is paving the way for small, stable, and user-friendly reference electrodes that can support next-generation portable and wearable sensors.

## Figures and Tables

**Table 1:** Comparison of reference membrane architectures reported in the recent literature.

Architecture	Membrane Composition and Contact	Mechanism of Potential Stabilization	Reported Stability/Drift	Typical Application	Reference
Liquid-junction-free polymeric membrane	PVC membrane doped with organic electrolyte (Q^+^B^−^)	Self-buffered ion partitioning at membrane/solution interface	ΔE < 1 mV over 24 h	Miniaturized solid-state sensors	[11]
Ionic-liquid junction with 3D carbon solid contact	PVC + ionic liquid (C_8_mimNTf_2_) + 3DOM carbon	Ionic liquid forms self-contained ionic junction	42 µV h^−1^ over 26 days	Long-term analytical sensing	[12]
PEDOT solid contact + ionogel capping	PEDOT:PSS + ionogel (ionic liquid in silica matrix)	High-capacitance transducer + diffusion barrier	<0.5 mV day^−1^	Wearable and flexible sensors	[13]
Carbon nanotube solid-state RE	CNT film + PVC/KCl membrane	Capacitive charge buffering and low resistance path	Stable ± 1 mV (12 h)	Disposable sensor arrays	[3]
Ag/Ag-organic salt SCRE	Ag/AgTCl_n_ (insoluble salt) + hydrophobic polymer layer	Stable solid electrolyte, minimized leakage	Drift < 0.2 mV h^−1^	Potentiometric ion sensing	[14]
Silicone-based IL-doped membrane	Biocompatible silicone + IL ([BMIM][NTf_2_])	IL provides internal ionic conductivity, no leakage	<1 mV over 8 h	Implantable biosensors	[15]
Polymer-based reference membranes	PVC, PU, and PMMA membranes with salt additives	Ion buffering and limited leaching	<1 mV drift in stable media	General-purpose solid-state RE	[1]

**Table 2:** Functional roles of components in the polymer membrane of reference electrodes.

Membrane Component	Function	Examples	References
Polymer	Matrix providing mechanical stability	PVC, PU	[20,21]
Plasticizer	Improving flexibility, mechanical stability, and ion mobility	o-NPOE, DOS	[5,22]

**Table 3:** Techniques and methods for reference electrode assessment.

Technique/Method	Principle	Provided Information	References
OCP (Open Circuit Potential)	Measurement of the electrode potential in a solution without current flow	Potential drift over time, stability in different ion concentrations and pH	[4,44]
CP (Chronopotentiometry)	Measurement of the electrode potential in a solution with current flow	Polarizability, capacitance of the contact layer, susceptibility to drift	[4,49]
EIS (Electrochemical Impedance Spectroscopy)	Impedance measurement	Chemical and physical stability of the solid-contact layer, material degradation	[4,50]
Cyclic voltammetry (CV)	Monitoring the half-wave potential (E_1_/_2_) of a reversible redox couple	Changes in E_1_/_2_ over repeated scans indicate instability	[51,52]

**Table 4:** Examples of analytes detected using potentiometric sensing platforms incorporating membrane-based reference electrodes.

Analyte	Application/Context	Reference
Nitrate (NO_3_^−^)	Soil and water monitoring	[62]
Sodium (Na^+^)	Paper-based ion detection	[55]
Potassium (K^+^)	Paper-based or wearable ion detection	[15,55]
Chloride (Cl^−^)	Sweat analysis	[14]
Lead (Pb^2+^)	Potentiometric titrations	[57]
pH	Potentiometric titrations	[57]
Perchlorate (ClO_4_^−^)	Testing medium and environmental relevance	[32]

## Data Availability

No new data were created or analyzed in this study. Data sharing is not applicable to this article.

## Abbreviations

The following abbreviations are used in this manuscript:

ABS	Acrylonitrile butadiene styrene
Ag/AgCl	Silver/silver chloride
AgTPB	Silver tetraphenylborate
BMIM	1-Butyl-3-methylimidazolium
[C_8_mim][NTf_2_]	1-Methyl-3-octylimidazolium bis(trifluoromethylsulfonyl)imide
CIM	Colloid-imprinted mesoporous (carbon)
CNT	Carbon nanotube
CP	Chronopotentiometry
DETH	Diethylhexyl terephthalate
DMPP	2,2-Dimethoxy-2-diphenylacetophenone
DOS	Dioctyl sebacate
EGDMA	Ethylene glycol dimethacrylate
EDS	Energy-dispersive spectroscopy
EIS	Electrochemical impedance spectroscopy
[emim][FAP]	1-ethyl-3-methyl imidazolium tris(pentafluoroethyl)- trifluorophosphate
[emim][NTf2]	1-Ethyl-3-methylimidazolium bis(trifluoromethylsulfonyl)imide
ETH 500	Tetradodecylammonium tetrakis(4-chlorophenyl)borate
GC	Glassy carbon
HDDA	1,6-Hexanediol diacrylate
IL	Ionic liquid
KCl	Potassium chloride
KTpClPB	Potassium tetrakis(4-chlorophenyl)borate
KTPhB	Potassium tetraphenylborate
MOF	Metal–organic framework
MSP	Multi-solution protocol
NaCl	Sodium chloride
NaClO_4_	Sodium perchlorate
NaNO_3_	Sodium nitrate
Na_2_SO_4_	Sodium sulfate
NDPE	2-nitrophenyl decyl ether
NPOE/o-NPOE	2-Nitrophenyl octyl ether
NTf_2_	Bis(trifluoromethylsulfonyl)imide
OCP	Open circuit potential
PBV	Poly(vinyl butyral)
PDMS	Polydimethylsiloxane
PEDOT	Poly(3,4-ethylenedioxythiophene)
PET	Polyethylene terephthalate
PMMA	Poly(methyl methacrylate)
POT	Poly(3-octylthiophene-2,5-diyl)
PSS	Poly(styrene sulfonate)
PU	Polyurethane
PVC	Poly(vinyl chloride)
Py	Polypyrrole
QRE	Quasi-reference electrode
RE	Reference electrode
SCRE	Solid-contact reference electrode
SEM	Scanning electron microscope
SPE	Screen-printed electrode
SSC	Solid-state composite
SWCNT	Single-walled carbon nanotube
TBA-TBB	Tetrabutylammonium tetrabutylborate
TBATPB	Tetrabutylammonium tetraphenylborate
TMACl	Tetramethylammonium chloride
UV	Ultraviolet
3DOM	Three-dimensionally ordered macroporous (carbon)
